# TMJ imaging in orthodontic patients presenting with facial pain and malocclusion: A radiological-clinical correlation study

**DOI:** 10.6026/973206300221207

**Published:** 2026-02-28

**Authors:** Vasanthan Palanivel, Shweta Neha Lakra, Sunitha Kesidi, Rachana Negi, Sushma Darnal, Nazish Akhtar

**Affiliations:** 1 Department of Orthodontics and Dentofacial Orthopaedics, Vinayaka Missions Sankarachariyar Dental College, Salem, Tamil Nadu, India; 2 Department of Orthodontics & Dentofacial Orthopaedics, Ranchi, Jharkhand, India; 3 Department of Dentistry, Government Medical College and Hospital, Narsampet, Telangana, India; 4 Department of Oral Medicine & Radiology, Seema Dental College & Hospital, Rishikesh, Uttarakhand, India; 5 Department of Oral Medicine & Radiology, I.T.S Centre for Dental Studies and Research, Ghaziabad, Uttar Pradesh, India

**Keywords:** Facial pain, malocclusion, radiology, TMJ dysfunction, treatment

## Abstract

Temporomandibular joint (TMJ) disorders are a common cause of facial pain and their association with malocclusion in orthodontic patients remains inadequately explored. Therefore, it is of interest to investigate the correlation between malocclusion, TMJ dysfunction and facial pain in 100 orthodontic patients. The results revealed that Class II and Class III malocclusions were significantly associated with more severe TMJ dysfunction and pain. Radiological findings such as condyle deformities, joint space narrowing and disc displacement were observed in the majority of patients. This study advances our knowledge by highlighting the importance of early diagnosis and multi- modal imaging in managing TMJ disorders in orthodontic populations.

## Background:

Temporomandibular joint (TMJ) disorders are a significant cause of facial pain and dysfunction, often associated with malocclusion and various orthodontic conditions. The TMJ plays a pivotal role in the proper function of the masticatory system, with any alteration in its structure or function leading to discomfort, pain and compromised oral health [1]. Among the most common symptoms of TMJ disorders are facial pain, limited jaw movement, clicking and popping sounds during mandibular movement. These symptoms are frequently observed in orthodontic patients who present with malocclusion, a condition where the alignment of the teeth and jaws is abnormal [2]. Malocclusion itself is a multifactorial condition, with genetic, environmental and functional influences contributing to its development. It is often associated with irregularities in the alignment of the teeth and the position of the jaws, which can place undue stress on the TMJ and surrounding structures [3]. This misalignment may lead to both acute and chronic TMJ dysfunction, often manifesting as facial pain and discomfort. Additionally, malocclusion, particularly in cases involving severe bite misalignment, has been correlated with an increased risk of developing TMJ disorders due to altered mandibular movements and occlusal stresses [4].

Radiographic imaging is critical in evaluating TMJ disorders and understanding their correlation with facial pain and malocclusion. Among the various imaging modalities, panoramic radiographs, cone-beam computed tomography (CBCT) and magnetic resonance imaging (MRI) are frequently used in the assessment of the TMJ and surrounding structures [5]. These techniques allow for a detailed examination of the bony and soft tissue components of the joint, including the condyle, articular disc and the surrounding ligaments and muscles. MRI, in particular, provides detailed images of soft tissues, including the articular disc, making it an invaluable tool in diagnosing internal derangements of the TMJ [6]. The relationship between facial pain, malocclusion and TMJ abnormalities has been well documented, but the exact pathophysiology remains unclear. Several studies suggest that the severity of malocclusion, including factors such as overjet, overbite and crossbite, may be linked to an increased risk of TMJ dysfunction [7]. However, the clinical presentation of TMJ disorders in orthodontic patients is complex and may vary based on the individual's orthodontic condition, the severity of malocclusion and the type of TMJ disorder present [8]. The diagnostic challenge lies in distinguishing between pains originating from the TMJ versus other causes, such as dental or muscular conditions, which can complicate treatment planning for orthodontic patients. Therefore, it is of interest to describe the radiological-clinical correlation in diagnosing TMJ disorders in patients with facial pain and malocclusion, which may enhance understanding and treatment planning.

## Methodology:

This study utilized a cross-sectional design to investigate the radiological-clinical correlation between TMJ disorders and malocclusion in orthodontic patients presenting with facial pain. The study was conducted at a tertiary care orthodontic clinic, where patients seeking orthodontic treatment or presenting with facial pain and malocclusion were recruited.

## Methodology outlines the steps involved in the study:

## Study population and sample size:

The study included orthodontic patients aged 18-45 years, who were diagnosed with malocclusion and presented with facial pain associated with TMJ disorders.

Patients were selected based on the following inclusion criteria:

[1] Patients with a clinically confirmed diagnosis of malocclusion

[2] Patients who reported facial pain or discomfort related to the TMJ

[3] Patients without any systemic or psychiatric disorders that may have affected the study outcomes

[4] Patients who were willing to participate in the study and provided written informed consent

A total of 100 patients were included in the study to ensure statistical power. The sample size was determined based on the estimated prevalence of TMJ disorders in orthodontic patients and a power calculation to detect significant correlations.

## Clinical evaluation:

Each patient underwent a comprehensive clinical evaluation, including:

[1] Facial Pain Assessment: Patients completed a detailed questionnaire to assess the severity, location and duration of facial pain. The Visual Analog Scale (VAS) was used to measure pain intensity.

[2] TMJ Dysfunction Index (TDI): This index was used to assess the presence of TMJ symptoms such as clicking, popping, or locking.

[3] Malocclusion Classification: Patients were categorized according to the Angle classification of malocclusion (Class I, II, or III) and further examined for specific occlusal discrepancies such as overbite, overjet and cross bite.

[4] Range of Jaw Movement: Jaw mobility, including mouth opening and lateral movements, was measured to assess any limitations or deviations in mandibular function.

## Radiological assessment:

The study utilized various imaging modalities to assess the TMJ and its components:

[1] Panoramic Radiograph: A baseline panoramic radiograph was obtained for all patients to evaluate the overall structure of the TMJ, including condylar morphology and joint space.

[2] CBCT: High-resolution CBCT scans were performed to assess the bony structures of the TMJ in detail, including the condyle, articular eminence and the mandibular fossa. CBCT was also used to evaluate joint degeneration or deformities that may have contributed to TMJ dysfunction.

[3] MRI: It was used to assess soft tissues, particularly the articular disc and to identify any internal derangements such as disc displacement, degeneration, or perforation.

## Data collection:

Data was collected through the clinical evaluations and radiological assessments. The clinical and radiological findings were recorded separately and then correlated to evaluate the relationship between malocclusion, facial pain and TMJ disorders. The clinical data included pain intensity (measured using the VAS), TMJ dysfunction (assessed using TDI) and malocclusion classification. The radiological data included the presence of any structural abnormalities, degenerative changes, or soft tissue disorders detected through imaging

## Statistical analysis:

Data was analyzed using appropriate statistical methods. Descriptive statistics (mean, standard deviation) were used to summarize clinical and radiological findings. The relationship between clinical symptoms (facial pain, malocclusion, jaw movement restrictions) and radiological findings (TMJ abnormalities, condylar degeneration, disc displacement) was assessed using correlation analysis (Pearson's or Spearman's correlation, depending on the data distribution). Multiple regression analysis was conducted to determine the strength of the relationship between malocclusion severity and TMJ disorders while controlling for potential confounding variables such as age, gender and treatment history. A p-value of < 0.05 was considered statistically significant.

## Ethical considerations:

The study adhered to ethical guidelines set by the institutional review board (IRB). Informed consent was obtained from all participants and patient confidentiality was maintained throughout the study. All patients were informed about the purpose of the study and their right to withdraw at any time without penalty.

## Results:

The results of the study were based on the analysis of both clinical and radiological data, including a total of 100 orthodontic patients who presented with facial pain and malocclusion. The data was collected through clinical evaluations, including the assessment of facial pain, TMJ dysfunction and malocclusion, as well as radiological evaluations using panoramic radiographs, CBCT scans and MRI. Below is the presentation of the results, including the statistical analysis, tables and findings (Table 1). The study included 100 participants, with a slight majority of females (55%) compared to males (45%). The average age of participants was 27.3 years. Malocclusion on was classified into three types: Class I (40%), Class II (30%) and Class III (30%). The facial pain intensity, as measured by the VAS, showed that 50% of patients reported moderate pain, 30% experienced mild pain and 20% had severe facial pain. This indicates a significant prevalence of moderate pain in the sample (Table 2). The TMJ dysfunction assessment using the TDI revealed that 40% of participants had mild dysfunction, while 35% experienced severe dysfunction. Only 25% of participants exhibited normal TMJ function, indicating a high prevalence of TMJ dysfunction among the sample population (Table 3). Class I malocclusion had the highest percentage of patients with normal TMJ function (37.5%) and the lowest percentage of severe dysfunction (25%). Class II and Class III malocclusion types exhibited a higher prevalence of severe TMJ dysfunction (43.3% and 40%, respectively) (Table 4. The panoramic radiographs showed that 40% of patients had condyle deformities and 25% had joint space narrowing, suggesting structural abnormalities in the TMJ. Interestingly, 35% of patients had no significant findings, highlighting the diversity in the radiological presentation of TMJ disorders (Table 5).

MRI scans revealed that 50% of patients had normal disc positioning, while 35% exhibited disc displacement and 15% showed signs of disc degeneration. This suggests that disc displacement is a common finding in patients with facial pain and malocclusion (Table 6). The analysis demonstrated a strong correlation between TMJ dysfunction and facial pain intensity (r = 0.72, p < 0.05), indicating that higher levels of dysfunction were associated with more intense facial pain. Furthermore, radiological findings of condyle deformities and joint space narrowing were more common in patients with severe TMJ dysfunction. The study also highlighted that patients with Class II and Class III malocclusions were more likely to experience TMJ dysfunction and associated pain compared to those with Class I malocclusion.

## Discussion:

In this cross-sectional radiological-clinical correlation study of 100 orthodontic patients presenting with facial pain and malocclusion, we observed a high prevalence of TMJ abnormalities on imaging that correlated with clinical dysfunction and pain intensity. On clinical assessment, moderate to severe facial pain was reported by the majority of patients and TMJ dysfunction was prevalent across all malocclusion types. Imaging revealed a variety of osseous and soft tissue changes, including condylar deformities and disc displacement. These findings are consistent with, but extend the literature in several key ways when compared to previous PubMed indexed research. The foundational study by Hans *et al.* (1992) [9] compared clinical examination, patient history and MRI findings in juvenile orthodontic patients and found that MRI identified anterior disc displacement in 11.8% of subjects, often without corresponding clinical signs, highlighting that imaging can detect occult internal derangements missed clinically. Our study expands upon these results by using both CBCT and MRI and we found a substantially higher prevalence of structural abnormalities, particularly in adult patients with more severe pain and dysfunction. This suggests the importance of combined imaging modalities in older, symptomatic populations. In a comparative evaluation of TMJ imaging modalities, CBCT demonstrated superior diagnostic accuracy for detecting bony changes compared with MRI in TMJ disorders, with CBCT showing higher sensitivity and specificity. Our results parallel this finding, as CBCT was particularly effective at identifying osseous abnormalities (e.g., condylar deformity, joint space narrowing), which also correlated with severe clinical dysfunction and pain in our cohort. MRI, meanwhile, was valuable for detecting soft tissue changes such as disc displacement in a significant proportion of patients [10]. The study by John *et al.* (2020) [11] using MRI to compare articular disc position among malocclusion types demonstrated that Class II vertical cases had more pronounced MRI detected TMJ morphological changes compared to other malocclusions. This aligns with our findings where Class II and Class III malocclusions exhibited higher rates of severe dysfunction and morphological abnormalities on imaging compared to Class I.

Both sets of findings suggest that specific malocclusion patterns may predispose patients to internal derangements that are better characterized with advanced imaging. The recent meta-analysis on TMD prevalence in patients with malocclusion showed that approximately 43% of malocclusion patients have TMD, with higher prevalence in Class II and crossbite subtypes and a greater burden in adults and females. Our clinical data similarly showed higher dysfunction and pain scores among females and in Class II/III malocclusion groups, reinforcing the idea that demographic and occlusal variables influence TMJ pathology [12]. The national epidemiological study by Sim and colleagues found that history of orthodontic treatment was associated with TMJ clicking but not with TMJ pain or functional impairment after adjusting for age and sex. While our study did not specifically analyze treatment history, our findings support the value of detailed symptom assessment and multi modal imaging to detect structural changes that might not manifest as pain initially but may progress to functional impacts if unrecognized [13]. Overall, the results demonstrate that in orthodontic patients with facial pain and malocclusion, combined radiological assessment (CBCT + MRI) detects a broad spectrum of TMJ abnormalities that correlate with clinical dysfunction. These findings are broadly consistent with prior PubMed indexed research while emphasizing the importance of comprehensive imaging protocols and nuanced interpretation in symptomatic orthodontic populations. Continuous integration of clinical symptoms with advanced imaging improves the understanding and management of TMJ disorders in the setting of malocclusion.

## Conclusion:

We show the strong correlation between malocclusion severity and TMJ dysfunction, particularly in Class II and Class III patients. Radiological findings of condyle deformities, joint space narrowing and disc displacement emphasize the structural basis of TMJ disorders. Early diagnosis and intervention are crucial in preventing long-term complications in orthodontic patients.

## Figures and Tables

**Table 1:** Demographic data of participants

**Parameter**	**Value**
Age Range	18-45 years
Mean Age	27.3 years
Gender	
- Male	45 (45%)
- Female	55 (55%)
Malocclusion Type	
- Class I	40 (40%)
- Class II	30 (30%)
- Class III	30 (30%)

**Table 2:** Facial pain assessment results

**Pain Intensity (VAS)**	**Frequency**	**Percentage (%)**
Mild (1-3)	30	30%
Moderate (4-6)	50	50%
Severe (7-10)	20	20%

**Table 3:** TDI results

**TDI Category**	**Frequency**	**Percentage (%)**
Normal Function	25	25%
Mild Dysfunction	40	40%
Severe Dysfunction	35	35%

**Table 4:** Correlation between malocclusion type and TMJ dysfunction

**Malocclusion Type**	**Normal Function**	**Mild Dysfunction**	**Severe Dysfunction**
Class I	15 (37.5%)	15 (37.5%)	10 (25%)
Class II	5 (16.7%)	12 (40%)	13 (43.3%)
Class III	5 (16.7%)	13 (43.3%)	12 (40%)

**Table 5:** Radiological findings from panoramic radiographs

**Radiological Finding**	**Frequency**	**Percentage (%)**
Condyle Deformity	40	40%
Joint Space Narrowing	25	25%
No Significant Findings	35	35%

**Table 6:** MRI findings of articular disc displacement

**Disc Position**	**Frequency**	**Percentage (%)**
Normal Position	50	50%
Disc Displacement	35	35%
Disc Degeneration	15	15%
